# Comparative Trends in Human and Veterinary Antimicrobial Consumption in the European Union, 2019–2024

**DOI:** 10.3390/antibiotics15070664

**Published:** 2026-07-07

**Authors:** Telma de Sousa, Tiago Bugarim, Gilberto Igrejas, Patricia Poeta

**Affiliations:** 1 MicroART—Antibiotic Resistance Team, Department of Veterinary Sciences, University of Trás-os Montes and Alto Douro, 5000-801 Vila Real, Portugal; telmaslsousa@hotmail.com (T.d.S.); gigrejas@utad.pt (G.I.); 2 Functional Genomics and Proteomics Unit, University of Trás-os-Montes and Alto Douro, 5000-801 Vila Real, Portugal; 3 Associated Laboratory for Green Chemistry, University NOVA of Lisbon, 1099-085 Caparica, Portugal; 4 Department of Veterinary Medicine, University of Bari Aldo Moro, 70010 Valenzano, Italy; t.bugarim@phd.uniba.it; 5 Department of Genetics and Biotechnology, University of Trás-os-Montes and Alto Douro, 5000-801 Vila Real, Portugal; 6 CECAV—Veterinary and Animal Research Centre, University of Trás-os-Montes and Alto Douro, 5000-801 Vila Real, Portugal; 7 AL4AnimalS —Veterinary and Animal Research Centre, Associate Laboratory for Animal and Veterinary Science (AL4AnimalS), University of Trás-os-Montes and Alto Douro, 5000-801 Vila Real, Portugal

**Keywords:** antimicrobial resistance (AMR), one health, antibiotic consumption, European Union, veterinary medicine, public health, surveillance

## Abstract

Antimicrobial resistance (AMR) is a global health crisis addressed through a One Health framework. However, recent European Union (EU) surveillance data reveals a marked divergence in progress between the human and animal sectors. This study analyzes the most recent monitoring reports (European Surveillance of Antimicrobial Consumption Network and European Sales and Use of Antimicrobials for Veterinary Medicine, 2024) to compare the effectiveness of mitigation strategies across sectors. The findings expose a clear paradox: while the veterinary sector has achieved a structural 24.3% reduction in antimicrobial sales in the EU since 2018, human medicine has recorded a 2% increase in overall consumption, diverging from established reduction targets. From a qualitative perspective, veterinary medicine has nearly eliminated the use of critically important antimicrobials in the AntiMicrobial Expert Group (AMEG) (category B), including polymyxins and third-generation cephalosporins, which now account for only 0.24% of total sales. In contrast, human medicine continues to struggle to contain antimicrobial resistance in key sentinel pathogens, notably *Klebsiella pneumoniae* and *Escherichia coli.* Furthermore, companion animals, representing 97.9% of non-food-producing animal biomass, emerge as a reservoir of antimicrobial-resistant bacteria due to the intensive use of broad-spectrum oral formulations. The results indicate that the veterinary regulatory model, centered on binding volume reduction and preventive strategies, has been more effective in reducing overall antimicrobial consumption compared to the voluntary, guideline-based stewardship approaches currently used in human medicine. Achieving meaningful control of antimicrobial resistance will require human medicine to adopt the same level of structural rigor already implemented in animal production systems.

## 1. Introduction

### Institutional Framework and Global Surveillance Networks for Combating AMR

Antimicrobial resistance (AMR) persists as a critical threat to global health [1]. Despite recognition of the problem, the implementation of effective measures often faces political inertia and operational challenges in various sectors, delaying the necessary response. To combat this crisis in a coordinated manner, the One Health approach was adopted, led by a network of strategic entities that provide the data analyzed in this review [2]. At the global level, the strategy is guided by the WHO (World Health Organization), which monitors the clinical impact and antimicrobial resistance rates through the GLASS [3], and by the WOAH (World Organisation for Animal Health), which oversees global veterinary health standards (Table 1). On the other hand, at the European level (EU triad), the ECDC (European Centre for Disease Prevention and Control) is responsible for epidemiological surveillance in human health [4]. The European Medicines Agency (EMA) manages the European Surveillance of Antimicrobial Consumption Network (ESAC-Net), a source of community and hospital consumption data that indicates current trends [4]. EMA regulates the pharmaceutical market and monitors the veterinary sector through the European Sales and Use of Antimicrobials for Veterinary Medicine (ESUAvet) project [5]. It is also responsible for the AMEG (AntiMicrobial Expert Group) categorization, defining which antibiotics should be restricted in animals to protect human health [6]. EFSA (European Food Safety Authority), in turn, acts at the interface of food safety, monitoring the presence of resistant bacteria in the food chain. The European Commission also plays an active role, defining policy targets (such as the Farm to Fork strategy) and binding legislation [7].

## 2. Data Sources and Methodology

This study conducted a comparative analysis of antimicrobial consumption and antimicrobial resistance trends in the European Union and European Economic Area (EEA) between 2019 and 2024. Data concerning human health was retrieved from the European ECDC surveillance reports, specifically through the European Surveillance of Antimicrobial Consumption Network (ESAC-Net). Veterinary data were obtained from the European Medicines Agency (EMA) via the ESUAvet project. Additionally, the global context and antimicrobial resistance metrics were supplemented by data from the World Health Organization’s Global Antimicrobial Resistance and Use Surveillance System (GLASS) 2025 report.

To compare sectors with distinct surveillance frameworks, specific metrics were utilized as proxies for selective pressure. Human consumption is expressed in defined daily doses (DDDs) per 1000 inhabitants per day, reflecting standardized therapeutic intensity in both community and hospital settings. In contrast, veterinary sales data are expressed in milligrams of active substance per Population Correction Unit (mg/PCU), normalized by animal biomass. While these units are not directly convertible, their relative temporal trends were analyzed to assess progress against the “Farm to Fork” strategy targets. Furthermore, the qualitative analysis of antibiotic use was based on the EMA’s AMEG categorization (Categories A to D) for veterinary medicine and the WHO AWaRe classification (Access, Watch, Reserve) for human medicine, focusing on the restriction of critically important antimicrobials such as third/fourth-generation cephalosporins, polymyxins, and carbapenems.

The comparative analysis was based solely on descriptive statistics to evaluate antimicrobial consumption patterns and temporal trends. No formal statistical hypothesis testing was performed due to the distinct nature and non-convertibility of the human and veterinary metrics used.

## 3. Global Surveillance Coverage and Data Representativeness

**Figure 1:**
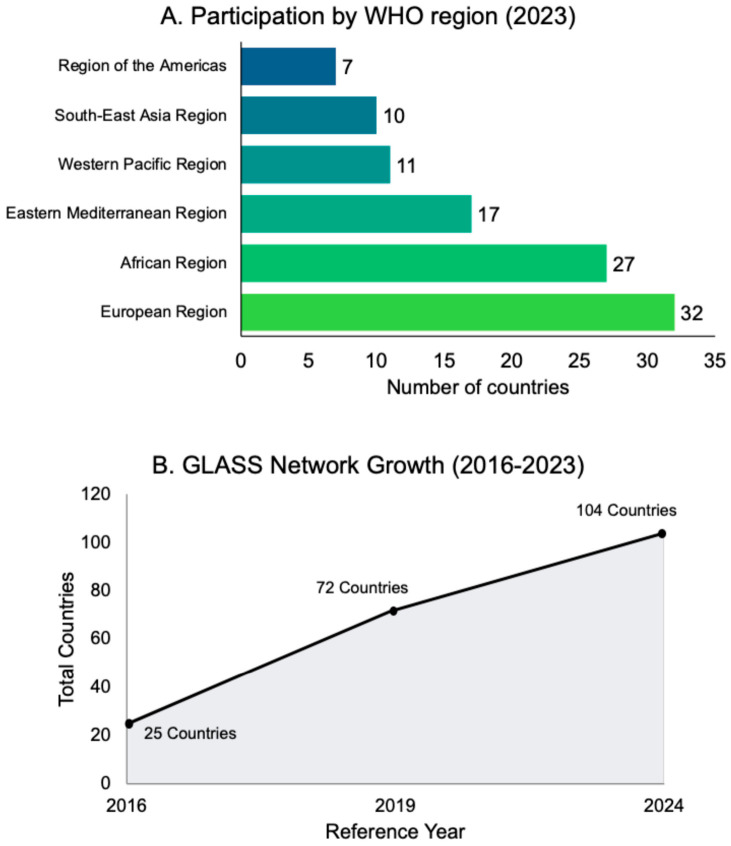
Global Participation in the GLASS Network (2016–2023). (**A**) Number of reporting countries in 2023 categorized by WHO Region, showing the highest participation in the European and African regions. (**B**) Annual growth of the GLASS network, illustrating a 316% increase in the number of countries reporting AMR data since the system’s launch. Adapted from [3].

The validity of the global analysis of antimicrobial resistance depends directly on the geographical representativeness of the data. The GLASS 2025 report shows remarkable progress in this area, as illustrated by the evolution of the number of reporting countries between 2016 and 2023 (Figure 1A) [3]. The validity of the conclusions of the GLASS 2025 report is based on an unprecedented sample base, comprising more than 23 million bacteriologically confirmed infections, reported by 104 countries (Figure 1B) [3,8].

A linear growth trend is observed in the number of countries and territories reporting antimicrobial resistance data [9,10,11]. The system, which began its activities with limited coverage in 2016, expanded substantially until 2023, progressively incorporating more low- and middle-income economies [12]. Although the European Region has historically maintained the highest and most consistent reporting rates (due to the maturity of networks such as EARS-Net) [13], the period from 2016 to 2023 stands out for its capacity-building efforts and integration of new countries in critical regions such as Africa and Southeast Asia. This expansion is vital to understand the “rebound” in antibiotic use observed after the initial disruptions of the COVID-19 pandemic [14].

To mitigate disparities in the quality of surveillance between different nations, WHO applied advanced statistical models to generate adjusted antimicrobial resistance estimates. This methodology focused on eight priority pathogens, including *Acinetobacter* spp., *Escherichia coli* (*E. coli*), *Klebsiella pneumoniae* (*K. pneumoniae*), *Neisseria gonorrhoeae*, non-typhoidal *Salmonella*, *Shigella* spp., *Staphylococcus aureus*, and *Streptococcus pneumoniae* [8]. Four infection types were considered: bloodstream (septicemia), gastrointestinal, urinary tract, and urogenital gonorrhea. The model weighted differences in population structure, national surveillance coverage, and the distribution of AMR among patient groups over time [11]. This statistical treatment resulted in the analysis of 93 combinations (infection type–pathogen–antibiotic), offering the most reliable picture to date of the antimicrobial resistance status at the regional, national and global levels. Furthermore, the report introduces a new “scoring framework” to assess the maturity of surveillance systems, encouraging countries to improve not only the quantity but also the representativeness of their data [15].

## 4. Data Analysis and Current Trends

When comparing the human and veterinary sectors from a One Health perspective, it is imperative to distinguish the surveillance metrics used. In human health (ECDC/ESAC-Net reports), data refers to ‘consumption’, expressed in DDD per 1000 inhabitants, a unit that reflects standardized therapeutic intensity [16]. In contrast, in animal health (EMA/ESUAvet and DGAV reports), monitoring is based on ‘sales’ data from marketing authorization holders, expressed in milligrams of active substance normalized by animal biomass (mg/PCU) [5,17].

Although these units (DDD vs. mg/PCU) are not directly convertible or comparable in absolute terms, the analysis of their relative trends over time is valid and robust [8,18,19]. Both serve as reliable proxies for the selective pressure exerted by antimicrobials in their respective ecosystems [20].

### 4.1. Human Sector and Stagnation and Recovery of Consumption

Even though the COVID-19 pandemic was expected to induce greater awareness regarding antibiotic use for infection control, the most recent data shows a return to pre-pandemic patterns [21,22]. In 2024, the weighted average of total consumption (community and hospital sectors combined) reached 20.3 DDD per 1000 inhabitants/day. This value represents a 2% increase compared to 2018 (the baseline year) [23]. This data is critical, as it places the European Union on a trajectory diverging from the target set for 2030, which foresees a 20% reduction in total consumption [24]. The temporal evolution confirms that the sharp drop observed in 2020–2021 was circumstantial (due to lockdowns and reduced circulation of respiratory viruses) and not structural [21,25]. With the return to social normality, consumption “rebounded” to levels higher than those of 2019. The report also highlights large disparities between countries, with values ranging from 9.8 DDD (in Nordic countries) to 29.9 DDD (in Southern Europe), showing that stewardship policies are not having uniform effectiveness [4,23].

The WHO’s Access, Watch, Reserve (AWaRe) classification of antimicrobials is a tool to assess and monitor antibiotic use and support antibiotic stewardship efforts, emphasizing the importance of the prudent use of antimicrobials (Table 2) [26]. The analysis is based on the most recent classification, published by the WHO in 2025. WHO AWaRe Access antimicrobials are mostly first- and second-line therapies that offer the best therapeutic value while minimizing the potential for antimicrobial resistance [27]. WHO AWaRe Watch antimicrobials have a broad spectrum of activity, and management efforts should limit their empirical use to severe infections or infections with bacteria that are more likely to be resistant to Access antimicrobials. WHO AWaRe Reserve antimicrobials include last-resort antimicrobials and should be reserved for the treatment of infections caused by multidrug-resistant organisms [26,27].

In 2024, first-line antibiotics (which should be the preferred choice) accounted for 60.3% of total consumption in the EU [4]. Although this figure technically meets the WHO’s minimum target (which recommends that at least 60% of total consumption should be from this category), the margin is narrow [24]. As with the total volume, the variability between countries is enormous, ranging from 38.6% (in countries with high use of broad-spectrum antibiotics) to 80.9% (in Nordic countries) [4]. The fact that almost 40% of European consumption still belongs to the “Watch” and “Reserve” categories indicates that, despite stabilization, there is an overuse of second-line substances that should be reserved, contrasting with the veterinary sector which has managed to reduce the use of critical antibiotics (category B) to residual levels [28].

To understand the severity of the surge in 2024, it is crucial to revisit the “bottom of the curve” recorded in 2020. During the first year of the COVID-19 pandemic, the European Union witnessed a historic drop in antimicrobial consumption, proving that a drastic reduction was possible, albeit for circumstantial reasons [4,29]. In 2020, total consumption in the EU (community and hospital) fell to 16.4 DDD per 1000 inhabitants/day. This represented a decrease of more than 18% compared to 2019 (19.9 DDD). It was the largest annual drop recorded in the history of the European surveillance network [4,21,30]. The reduction occurred almost exclusively in the community sector (primary care), driven by lockdowns, social distancing, and the use of masks, which drastically reduced the transmission of other respiratory infections (influenza, RSV) and, consequently, the prescription of antibiotics [14,25,29,31]. Comparing the 16.4 DDD of 2020 with the 20.3 DDD of 2024, it becomes evident that the “lesson” of hygiene and infection control has been forgotten. The health system did not capitalize on this structural decline, instead, it allowed an immediate return to old practices as soon as society reopened [4,14].

**Figure 2:**
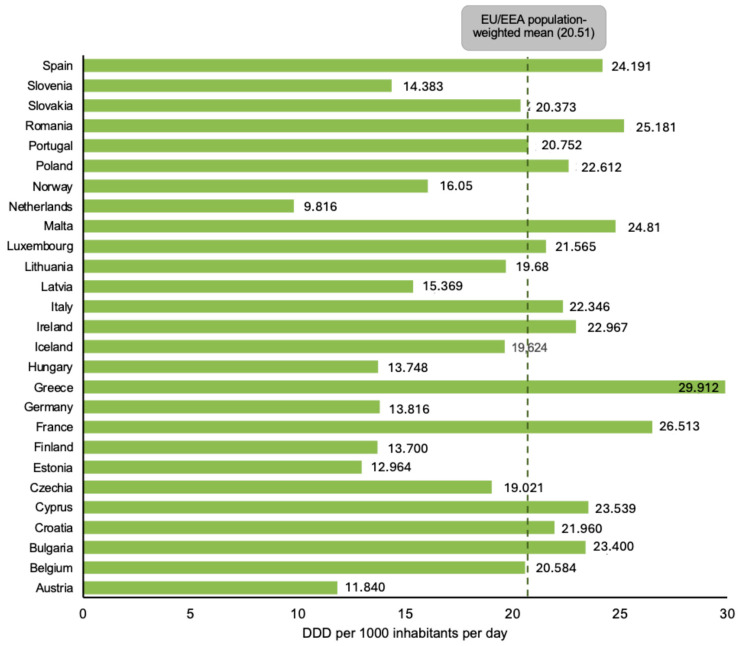
Total antimicrobial consumption in the EU/EEA in 2024 (DDD per 1000 inhabitants per day) and proportion of Access antibiotics by country, showing marked regional disparities. Adapted from [13].

Analysis of the human sector reveals a double concern: the volume of consumption has risen again, and the quality of prescribing (antibiotic choice) has steered away from European targets [32]. In 2024, the average total consumption in the EU/EEA was 20.51 DDD per 1000 inhabitants/day (Figure 2). This value represents a 2% increase compared to 2019, offsetting the temporary drop observed during the pandemic [21]. According to the WHO classification, at least 60% of total consumption should belong to the “Access” group [33]. In 2024, the EU average was only 60.3%, representing a 1.0 percentage point decrease compared to 2019. Currently, we are 4.7 percentage points away from the 2030 target (>65%), with no statistically significant improvement trend over the last 5 years. The European average masks serious regional disparities. While Iceland leads with 80.9% consumption in the “Access” category [13], Slovakia reports only 38.6%, indicating excessive use of broad-spectrum antibiotics (“Watch” and “Reserve” groups) in vast areas of Europe. Only 9 EU countries have reached the proposed quality target for 2030 [26,27,32].

### 4.2. Animal Sector and Structural Reduction in Sales

During 2025, 29 countries (27 EU countries plus Iceland and Norway) reported 2024 data to the European Medicines Agency (hereinafter referred to as the “Agency” or EMA) on the sales volume of veterinary antimicrobial medicinal products (VAMs) and on the use of antimicrobial medicinal products in animals, under Article 57 of Regulation (EU) 2019/6 [34]. The transition to species-level usage data represents a significant advance in understanding antimicrobial exposure in animals.

By comparison to the stagnation observed in human medicine, the veterinary sector shows a consolidated long-term downward trajectory, driven by the implementation of Regulation (EU) 2019/6 and the “Farm to Fork” strategy [19,35].

**Figure 3:**
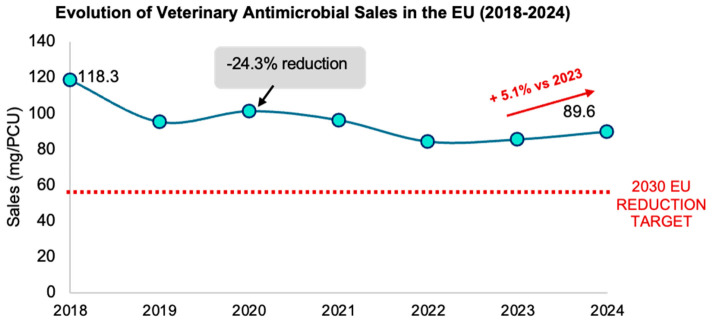
Evolution of EU veterinary antimicrobial sales and progress toward 2030 Targets. Sales volume is expressed in mg/PCU. The graph illustrates a 24.3% overall reduction from the 2018 baseline (118.3 mg/PCU) to 2024 (89.6 mg/PCU), despite a minor 5.1% uptick in 2024 (highlighted in red). The red dashed line represents the 2030 “Farm to Fork” target of 59.2 mg/PCU (50% reduction). Adapted from [5,18].

Analysis of harmonized sales data (mg/PCU) reveals that the European Union reduced antibiotic consumption in animals by 24.3% between 2018 (the base year of the strategy) and 2024, falling from 118.3 mg/PCU to 89.6 mg/PCU (Figure 3). However, the 2024 report by the EMA indicates a slight increase (+5.1% in mg/kg) compared to the previous year, suggesting that, after the initial sharp decline, the reduction curve may be stabilizing or experiencing market fluctuations.

**Figure 4:**
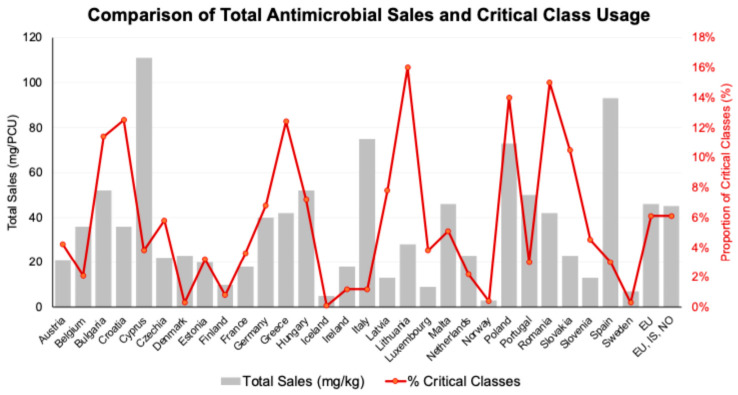
Comparison of total veterinary antimicrobial sales (mg/kg) across EU countries in 2024, highlighting the predominance of first-line Category D antibiotics, overlaid with the percentage of critical category B agents (third/fourth-gen cephalosporins, quinolones, polymyxins. Adapted from [5].

More important than volume reduction is the qualitative profile of the drugs used (Figure 4). In 2024, the distribution of sales in the EU demonstrated a clear alignment with the precautionary recommendations. Category D (“Precaution”), represented the vast majority of sales (67.6%, 31.1 mg/kg), confirming that first-line treatments (e.g., simple penicillins, tetracyclines) are the preferred choice [15]. Category B (“Restricted”), antibiotics of critical importance to human health (third/fourth-generation cephalosporins, quinolones and polymyxins) constituted only 6.0% (2.8 mg/kg) of total sales in the EU [5,34]. In 2024, the proportion of sales (in mg/kg) corresponding to each of the AMEG category B antimicrobials varied substantially between countries, ranging from 0% to 1.5% for third- and fourth-generation cephalosporins, <0.01% to 10.5% for fluoroquinolones, 0% to 6.7% for other quinolones, and 0% to 10.9% for polymyxins. Specifically, sales of third- and fourth-generation cephalosporins were negligible (0.24% of the total), highlighting the success of veterinary restriction policies in protecting these “life-saving” molecules.

The implementation of actual use data collection in the 2024 report marks a turning point in epidemiological surveillance, allowing for precise identification of the sectors exerting the greatest selective pressure. The data reveals that antibiotic pressure is not uniform. Pig and poultry farming continue to account for the largest share of consumption [36]. In terms of biomass (animal population), cattle (31.8%), pigs (26.0%), and poultry (14.8%) constitute most production animals in the EU. This pattern indicates that antibiotic pressure is disproportionately concentrated in short-cycle production systems [5,37,38].

**Figure 5:**
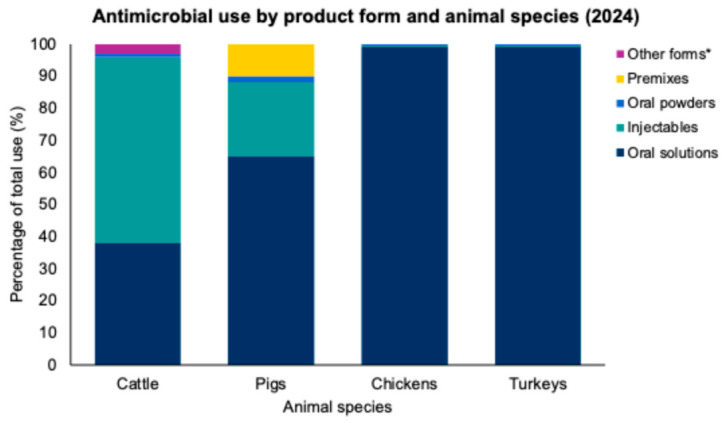
Comparison of antimicrobial use by animal species and product form in 2024. Data are expressed as a percentage of total use (measured in tons of active substance) for cattle, pigs, chickens, and turkeys. For cattle, “other forms” include intramammary and intrauterine products, oral pastes, and tablets. These figures represent consolidated data from countries with at least 90% use data coverage. Adapted from [5].

One of the most positive indicators is the change in the route of administration, reflecting a shift in veterinary practices. A consistent reduction in the use of antibiotics administered via feed or drinking water was observed (Figure 5). Oral solutions (drinking water) accounted for 18.3% and oral powders 8.9%. Although oral forms still account for approximately 53.3% of sales, their recorded decline is the main factor driving the overall decrease in antibiotic use in Europe [18,39]. This is crucial because the oral route is associated with group treatments (metaphylaxis), where sick and healthy animals are treated simultaneously [40]. Its reduction indicates a shift towards individual “targeted” treatment. The use of injectables remains stable or shows slight relative increases (accounting for 13.3% of total sales) [5]. While this may seem negative, in a One Health context this is often a sign of improvement: the veterinarian is treating the sick animal individually, rather than medicating the entire herd [41].

The report also highlights a frequently overlooked aspect in macro-level analysis: the epidemiological role of pets [42]. Although the total sales volume for this sector is small compared to intensive livestock farming, dogs and cats represent an overwhelming 97.9% of the estimated biomass in the “other animals” category (approximately 1.9 million tons), relegating fur-producing animals to a residual expression (2.1%) [5]. The central concern in this group lies not in the quantity, but in the quality of prescriptions; the intensity of use of Critically Important Antibiotics (CIAs) is disproportionately high [43]. The frequent use of oral formulations containing classes such as amoxicillin combined with clavulanic acid or fluoroquinolones effectively creates a reservoir of antimicrobial resistance “within the home” [43,44,45]. Given the close coexistence and shared environment between these animals and their owners, a direct route of transmission of multidrug-resistant bacteria is established, extending a risk previously mainly associated with agricultural settings to intimate family contact [44].

## 5. EU Antimicrobial Sales Reduction Target and Impact on Bacteriological Profile

The European strategy is not limited to reducing volumes; it establishes a direct correlation between decreased sales and the control of sentinel bacteria (“bug–drug combinations”) that represent the greatest threat to public health [46].

The target of reducing human antibiotic consumption by 20% is not arbitrary, it aims to alleviate selective pressure on three critical outcome indicators for 2030 (Table 3). However, the reported increase in consumption in 2024 (+2% compared to 2019) directly jeopardizes these clinical objectives. Regarding the threat of *K. pneumoniae* (Target: −5% carbapenem resistance), this is the most alarming situation. Carbapenems are “reserve” antibiotics (a hospital’s last resort) under the WHO classification [47]. The inability to contain hospital consumption and cross-transmission has led to a trend of increasing antimicrobial resistance in several Member States, instead of the desired 5% reduction [48]. Global surveillance data confirm that carbapenem-resistant Enterobacterales (CRE) detection increased significantly in the European region between 2018 and 2022, and mathematical models demonstrate that without substantial reductions in consumption, antimicrobial resistance frequencies are unlikely to decline [48,49,50].

In the case of *E. coli* (Target: −10% resistance to third-generation cephalosporins), antimicrobial resistance is fueled by the overuse of “Watch” group antibiotics (such as Ceftriaxone) in the community and hospitals [8]. With the EU average missing the AWaRe quality target (only 60% “Access” use), selective pressure on *E. coli* remains high, hindering the 10% reduction in the incidence of resistant bloodstream infections [24].

Methicillin-resistant *Staphylococcus aureus* (MRSA) (Target: −15%), historically the “number one enemy”, shows variable trends. Although some countries show slight improvements (the result of decades of infection control), prevalence remains high in southern Europe [13].

**Figure 6:**
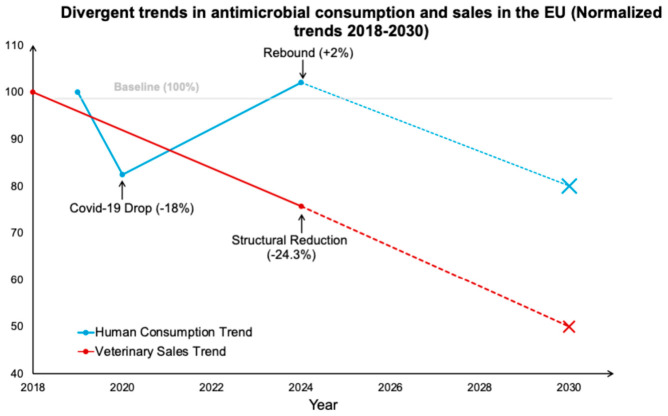
Divergent trends in antimicrobial consumption (human) and sales (veterinary) in the EU normalized to baseline years (2018–2030). Human consumption (blue line, 2019 baseline) shows a 2% increase, while veterinary sales (red line, 2018 baseline) show a 24.3% reduction. Dotted lines represent the linear trajectories required to achieve the 2030 reduction targets of 20% for human use and 50% for veterinary sales. Adapted from [6,13].

In the animal sector, the central objective, defined by the Farm to Fork strategy, is clear and binding, to reduce global sales of antimicrobials for livestock and aquaculture by 50% by 2030 (compared to the baseline year of 2018). The EU achieved a 24.3% reduction (from 118.3 to 89.6 mg/PCU), but the 2030 target is 59.2 mg/PCU (Figure 6) [6].

Although the main target is volume, the EU still monitors success through specific bacterial indicators in healthy animals (at slaughter), which act as sentinels of the risk to humans. One example is the surveillance of third-generation cephalosporin-resistant *E. coli* from production animals [5]. By eliminating the prophylactic use of antibiotics and restricting the use of category B antimicrobials (e.g., cephalosporins), the selective pressure for antimicrobial resistance is expected to decrease, resulting in a lower prevalence of resistant bacteria within the intestinal microbiota of animals [51]. The massive reduction in sales of these critical drugs (now only 0.24% of the total) has led to a decreasing trend of antimicrobial resistance in *E. coli* isolates from poultry and swine in most member states [5,52]. Reducing the colonization of swine by methicillin-resistant *S aureus* reflects an improvement in biosecurity and hygiene on farms, reducing the need for tetracyclines and beta-lactams (which co-select MRSA) [53]. Hence, reducing sales of category B substances also becomes an important goal in animal antibiotic use.

Despite the encouraging reductions observed in veterinary antimicrobial sales, important challenges remain. Considerable variability persists among EU Member States regarding antimicrobial consumption, implementation of stewardship programs, livestock production systems, and national regulatory approaches. Similarly, although overall human antimicrobial consumption has remained relatively stable, several countries have achieved substantial reductions through targeted stewardship interventions, whereas others continue to report higher levels of consumption. These findings highlight that progress is heterogeneous across Europe and that successful strategies may differ according to national healthcare and agricultural contexts.

## 6. Study Limitations

This study presents some limitations that should be considered when interpreting the results. First, the comparison between the human and veterinary sectors is based on distinct surveillance indicators. Antimicrobial consumption in human medicine is expressed in defined daily doses (DDD per 1000 inhabitants per day), while in veterinary medicine the data correspond to sales of active substances standardized by animal biomass (mg/PCU). These indicators reflect different concepts and are not directly comparable in absolute terms. Consequently, the comparison carried out in this work is based exclusively on the temporal evolution and relative trends of each sector, and not on equivalent quantitative values.

A second limitation stems from the differences between the surveillance systems used. Human data comes from the ESAC-Net network and represents actual antimicrobial consumption, while veterinary data from ESUAvet are predominantly based on drug sales, although the progressive introduction of usage data by species is improving the accuracy of veterinary surveillance. These methodological differences may influence the direct comparability between the sectors. Furthermore, this study has an ecological design, using aggregated data at the European level. As such, it is not possible to establish causal relationships between antimicrobial consumption and observed antimicrobial resistance trends, nor to infer associations at the individual, institutional, or national level. Resistance patterns result from the interaction of multiple factors, including infection prevention and control practices, circulation of bacterial clones, antimicrobial use in different contexts, and characteristics of animal health and production systems.

Finally, although the analysis suggests that regulatory strategies implemented in veterinary medicine have been more effective in reducing antimicrobial use than approaches currently applied in human medicine, this interpretation should be understood in the context of methodological differences in surveillance systems and the specificities of each sector.

## 7. Conclusions

The comparative analysis of the most recent European surveillance data highlights divergent trends in antimicrobial use between the human and veterinary sectors within the One Health framework. While veterinary antimicrobial sales have shown a sustained reduction since 2018, human antimicrobial consumption has remained stable or slightly increased, indicating different levels of progress toward the European Union’s 2030 targets. Firstly, the results suggest that binding regulatory approaches appear to have been associated with greater reductions The structural reduction of 24.3% in sales of veterinary antimicrobials in the EU was not an accident, but this likely reflects the implementation of Regulation (EU) 2019/6 and the Farm to Fork strategy. By focusing on reducing the total volume (−50%) and the near-total restriction of critical antibiotics (category B), the animal sector managed to protect essential molecules such as colistin and third/fourth-generation cephalosporins. In Europe, veterinary antibiotic use has declined sharply in recent years, largely due to stringent regulations and coordinated stewardship in animal production. Binding EU policies (e.g., bans on antibiotic growth promoters, stricter prescription rules) and national strategies have been implemented to curb use. As a result, sales of antibiotics for food-producing animals fell by over 50% from 2011 to 2022. In parallel, farmers and veterinarians have adopted preventive measures (vaccination, biosecurity, reduced prophylaxis) that help maintain animal health with less antimicrobial use. In contrast, human antibiotic consumption has not declined as dramatically. Factors such as aging populations (more elderly patients), increased healthcare interventions and a rebound in prescriptions after the COVID-19 pandemic likely contributed to stagnating or rising use. For example, EU antibiotic consumption rebounded in 2022 compared to 2020–21, and the use of broad-spectrum antibiotics in humans has been increasing. These divergent trends have important consequences: reduced veterinary antimicrobial use may lessen the spread of resistant bacteria from animals, but rising human use can accelerate resistance in human pathogens. Indeed, resistance remains persistently high in key human and animal pathogens (e.g., ampicillin-, tetracycline-resistant bacteria), and only combined reductions in both sectors have been shown to significantly lower resistance levels.

Finally, this article highlights a new and often overlooked battlefront: companion animals. Because dogs and cats contribute little or nothing to food production, yet are frequently treated with critically important antimicrobials, they may constitute an “indoor” reservoir of antimicrobial resistance that warrants urgent surveillance and stewardship.

In short, the reviewed veterinary data suggests that substantial reductions are achievable to decouple farm productivity from antimicrobial use. The challenge for the next decade lies in the ability of human medicine to adopt the same structural rigor it has imposed on its veterinary counterparts.

## Figures and Tables

**Table 1:** Institutional framework and surveillance networks involved in the monitoring and control of AMR within the One Health approach at global and European levels.

Entity	Level of Action	Main Network/Report	Activity and Focus in Combating AMR
WHO (OMS)	Global	GLASS	Monitors clinical antimicrobial resistance (therapeutic failure rates) in priority human pathogens (e.g., *E. coli*, *K. pneumoniae*).
WOAH	Global	Animal Health	Defines global animal health standards and collaborates with WHO on the animal component of the One Health strategy.
ECDC	European (Human)	ESAC-Net	Epidemiological surveillance of human antimicrobial consumption (community and hospital). Issues alerts on progress toward EU targets.
EMA	European (Veterinary)	ESUAvet	Regulates the medicines market and monitors veterinary sales. Defines AMEG categorization (which antibiotics should be restricted in animals).
EFSA	European (Food)	Zoonoses Reports	Monitors the presence of resistant bacteria in the food chain and in food-producing animals.
European Commission	European (Political)	Farm to Fork	Defines political strategy and binding legislation (e.g., target of a 50% reduction in veterinary antimicrobial sales by 2030).

**Table 2:** WHO AWaRe classification of antibiotics, including definitions, representative examples, and recommended targets for use to support antimicrobial stewardship. Adapted from [15].

Category	Definition	Common Antibiotics (Examples)	Target/Use
**ACCESS** (Access)	Narrow-spectrum antibiotics, lower risk of antimicrobial resistance and better safety profile. Should be the first choice.	Amoxicillin (and Amoxicillin + Clavulanate); Penicillin; Ampicillin; Cephalexin (1st-generation cephalosporin); Nitrofurantoin (urinary tract infections); Gentamicin.	Target: >65% of total consumption. Should always be available.
**WATCH** (Alert)	Broader spectrum or higher risk of antimicrobial resistance. Use should be monitored and limited to specific indications.	Azithromycin/Clarithromycin (macrolides); Ciprofloxacin/Levofloxacin (fluoroquinolones); Cefuroxime (2nd-generation cephalosporin); Ceftriaxone (third-generation cephalosporin).	Should be reduced. Main targets of antimicrobial stewardship programs.
**RESERVE** (Reserve)	“Last-resort” antibiotics. Life-saving when all other options fail. Highly restricted use.	Colistin; Linezolid; Ceftazidime-Avibactam; Tigecycline; Meropenem/Imipenem (carbapenems).	Only for confirmed multidrug-resistant infections.

**Table 3:** Key human and animal antimicrobial resistance and consumption indicators in the European Union, 2030 targets, status (2024), and associated biological impact.

Domain	Key Indicator (Target)	2030 EU Target	Current Status (2024)	Biological Rationale/Impact
**Human**	Total antimicrobial consumption	Reduce by 20%	+2% (Increase)	Selective pressure remains high in both community and hospital settings.
	*E. coli* resistant to third-generation cephalosporins	Reduce by 10%	Stagnant	Excessive use of “Watch” antibiotics prevents a downward trend.
	*K. pneumoniae* resistant to carbapenems	Reduce by 5%	Increasing	Failure in hospital infection control; critical risk.
	MRSA (*Staphylococcus aureus*)	Reduce by 15%	Stable	Slight improvements, but prevalence remains high in Southern Europe.
**Animal**	Total veterinary antimicrobial sales	Reduce by 50%	−24.3% (On track)	Structural reduction in selective pressure along the food chain.
	Sales of third/fourth-generation cephalosporins	Minimize	0.2% (Residual)	Direct protection of antibiotics critical for human medicine.
	Sales of polymyxins (colistin)	Minimize	Sharp decline	Preservation of a “last-resort” option for human Gram-negative infections.
	Indicator *E. coli* (intestinal) prevalence	Reduce prevalence	Decreasing	Direct correlation with lower antibiotic load in animal feed.

## Data Availability

Publicly available datasets were analyzed in this study. This data can be found in the ECDC (ESAC-Net) and EMA (ESUAvet) surveillance reports.

## References

[1] Murray C.J., Ikuta K.S., Sharara F., Swetschinski L., Robles Aguilar G., Gray A., Han C., Bisignano C., Rao P., Wool E. (2022). Global Burden of Bacterial Antimicrobial Resistance in 2019: A Systematic Analysis. Lancet.

[2] One Health Joint Plan of Action (2022–2026). https://repository.gheli.harvard.edu/repository/14273/.

[3] World Health Organization (2025). Global Antibiotic Resistance Surveillance Report 2025 WHO Global Antimicrobial Resistance and Use Surveillance System (GLASS).

[4] European Centre for Disease Prevention and Control (ECDC) (2024). Antimicrobial Consumption in the EU/EEA (ESAC-Net)—Annual Epidemiological Report for 2023.

[5] European Medicines Agency (2025). European Sales and Use of Antimicrobials for Veterinary Medicine: Annual Surveillance Report for 2024.

[6] Categorisation of Antibiotics for Use in Animals for Prudent and Responsible Use. https://cmvro.ro/files/download/antibiorezistenta/categorisation-antibiotics-use-animals-prudent-responsible-use_en.pdf.

[7] Farm to Fork Strategy—Food Safety—European Commission. https://food.ec.europa.eu/system/files/2020-05/f2f_action-plan_2020_strategy-info_en.pdf.

[8] World Health Organization (WHO). https://www.who.int/.

[9] Van Boeckel T.P., Gandra S., Ashok A., Caudron Q., Grenfell B.T., Levin S.A., Laxminarayan R. (2014). Global Antibiotic Consumption 2000 to 2010: An Analysis of National Pharmaceutical Sales Data. Lancet Infect. Dis..

[10] Roberts S.C., Zembower T.R. (2021). Global Increases in Antibiotic Consumption: A Concerning Trend for WHO Targets. Lancet Infect. Dis..

[11] Klein E.Y., Impalli I., Poleon S., Denoel P., Cipriano M., Van Boeckel T.P., Pecetta S., Bloom D.E., Nandi A. (2024). Global Trends in Antibiotic Consumption during 2016–2023 and Future Projections through 2030. Proc. Natl. Acad. Sci. USA.

[12] Otaigbe I.I., Elikwu C.J. (2023). Drivers of Inappropriate Antibiotic Use in Low- and Middle-Income Countries. JAC Antimicrob. Resist..

[13] AMC|European Centre for Disease Prevention and Control. https://qap.ecdc.europa.eu/public/extensions/AMC2_Dashboard/AMC2_Dashboard.html#eu-consumption-tab.

[14] Nandi A., Pecetta S., Bloom D.E. (2023). Global Antibiotic Use during the COVID-19 Pandemic: Analysis of Pharmaceutical Sales Data from 71 Countries, 2020–2022. EClinicalMedicine.

[15] Sulis G., Sayood S., Katukoori S., Bollam N., George I., Yaeger L.H., Chavez M.A., Tetteh E., Yarrabelli S., Pulcini C. (2022). Exposure to World Health Organization’s AWaRe Antibiotics and Isolation of Multidrug Resistant Bacteria: A Systematic Review and Meta-Analysis. Clin. Microbiol. Infect..

[16] Leung V., Langford B.J., Ha R., Schwartz K.L. (2021). Metrics for Evaluating Antibiotic Use and Prescribing in Outpatient Settings. JAC Antimicrob. Resist..

[17] Grave K., Torren-Edo J., Mackay D. (2010). Comparison of the Sales of Veterinary Antibacterial Agents between 10 European Countries. J. Antimicrob. Chemother..

[18] Home—WOAH—World Organisation for Animal Health. https://www.woah.org/en/home/.

[19] Home|Food and Agriculture Organization of the United Nations. https://www.fao.org/home/en.

[20] Kallen M.C., Prins J.M. (2017). A Systematic Review of Quality Indicators for Appropriate Antibiotic Use in Hospitalized Adult Patients. Infect. Dis. Rep..

[21] Högberg L.D., Vlahović-Palčevski V., Pereira C., Weist K., Monnet D.L., Strauss R., Catry B., Ivanov I., ESAC-Net Study Group, ESAC-Net Study Group Participants (2021). Decrease in Community Antibiotic Consumption during the COVID-19 Pandemic, EU/EEA, 2020. Eurosurveillance.

[22] Ventura-Gabarró C., Leung V.H., Vlahović-Palčevski V., Machowska A., Monnet D.L., Högberg L.D., Strauss R., Catteau L., Stoikov I., Payerl-Pal M. (2023). Rebound in Community Antibiotic Consumption after the Observed Decrease during the COVID-19 Pandemic, EU/EEA, 2022. Eurosurveillance.

[23] World Health Organization (2025). Surveillance of Antimicrobial Resistance in Europe, 2024 Data.

[24] Council Recommendation on Stepping up EU Actions to Combat Antimicrobial Resistance in a One Health Approach—Public Health. https://health.ec.europa.eu/publications/council-recommendation-stepping-eu-actions-combat-antimicrobial-resistance-one-health-approach_en.

[25] Sulis G., Batomen B., Kotwani A., Pai M., Gandra S. (2021). Sales of Antibiotics and Hydroxychloroquine in India during the COVID-19 Epidemic: An Interrupted Time Series Analysis. PLoS Med..

[26] World Health Organization The WHO AWaRe (Access, Watch, Reserve) Antibiotic Book. https://www.who.int/publications/i/item/9789240062382.

[27] Hsia Y., Sharland M., Jackson C., Wong I.C.K., Magrini N., Bielicki J.A. (2019). Consumption of Oral Antibiotic Formulations for Young Children According to the WHO Access, Watch, Reserve (AWaRe) Antibiotic Groups: An Analysis of Sales Data from 70 Middle-Income and High-Income Countries. Lancet Infect. Dis..

[28] European Medicines Agency (2023). Sales of Veterinary Antimicrobial Agents in 31 European Countries in 2022—Trends from 2010 to 2022—Thirteenth ESVAC Report.

[29] Hoti A., Sutej I., Jakupi A. (2025). Impact of the COVID-19 Pandemic on Antibiotic Prescriptions at the University Clinical Dentistry Center of Kosovo. Antibiotics.

[30] European Centre for Disease Prevention and Control (ECDC) (2021). ESAC-NET AER 2020—Antimicrobial Consumption in the EU/EEA.

[31] Ji W., Zhao Y., Du J., Zhao H., McIver D.J., Ye D., Yan K., Wei X., Fang Y. (2025). Determining the Impact of the COVID-19 Pandemic on the Consumption of Antibiotics in Shaanxi Province, China: An Interrupted Time-Series Analysis. Front. Public Health.

[32] European Centre for Disease Prevention and Control (ECDC) (2025). Antimicrobial Consumption in the EU/EEA (ESAC-Net)—Annual Epidemiological Report for 2024.

[33] Sharland M., Cappello B., Ombajo L.A., Bazira J., Chitatanga R., Chuki P., Gandra S., Harbarth S., Loeb M., Mendelson M. (2022). The WHO AWaRe Antibiotic Book: Providing Guidance on Optimal Use and Informing Policy. Lancet Infect. Dis..

[34] Schmerold I., van Geijlswijk I., Gehring R. (2023). European Regulations on the Use of Antibiotics in Veterinary Medicine. Eur. J. Pharm. Sci..

[35] European Centre for Disease Prevention and Control (ECDC), European Food Safety Authority (EFSA), European Medicines Agency (EMA) (2024). Antimicrobial Consumption and Resistance in Bacteria from Humans and Food-producing Animals. EFSA J..

[36] FVE—Federation of Veterinarians of Europe What Gets Measures, Gets Managed: First-Ever European-Wide Report on Veterinary Antibiotic Use Marks New Era. https://fve.org/what-gets-measures-gets-managed-first-ever-european-wide-report-on-veterinary-antibiotic-use-marks-new-era/.

[37] Ardakani Z., Canali M., Aragrande M., Tomassone L., Simoes M., Balzani A., Beber C.L. (2021). The European Union Summary Report on Antimicrobial Resistance in Zoonotic and Indicator Bacteria from Humans, Animals and Food in 2018/2019. EFSA J..

[38] Joosten P., Sarrazin S., Van Gompel L., Luiken R.E.C., Mevius D.J., Wagenaar J.A., Heederik D.J.J., Dewulf J., Graveland H., Schmitt H. (2019). Quantitative and Qualitative Analysis of Antimicrobial Usage at Farm and Flock Level on 181 Broiler Farms in Nine European Countries. J. Antimicrob. Chemother..

[39] Decundo J.M., Dieguez S.N., Martínez G., Amanto F.A., Pérez Gaudio D.S., Soraci A.L. (2025). The Vehicle of Administration and Prandial State May Reduce the Spectrum of Oral Broad-Spectrum Antibiotics (Oxytetracycline, Fosfomycin and Amoxicillin) Administered to Piglets: A Pharmacokinetic/Pharmacodynamic Approach. J. Vet. Pharmacol. Ther..

[40] Jourquin S., Debruyne F., Chantillon L., Lowie T., Boone R., Bokma J., Pardon B. (2024). Noninferiority Trial in Veal Calves on the Efficacy of Oxytetracycline and Florfenicol Treatment for Pneumonia Guided by Quick Thoracic Ultrasound. J. Dairy Sci..

[41] Collignon P.J., McEwen S.A. (2019). One Health—Its Importance in Helping to Better Control Antimicrobial Resistance. Trop. Med. Infect. Dis..

[42] Horodyska I., Kasperska P., Michalski K., Bubak J., Herman I., Miszczak M. (2025). Natural Microbiota of Dogs and Cats as a Source and Vector of Resistance Genes-Clinical Significance. Int. J. Mol. Sci..

[43] Joosten P., Ceccarelli D., Odent E., Sarrazin S., Graveland H., Van Gompel L., Battisti A., Caprioli A., Franco A., Wagenaar J.A. (2020). Antimicrobial Usage and Resistance in Companion Animals: A Cross-Sectional Study in Three European Countries. Antibiotics.

[44] Van Cleven A., Sarrazin S., de Rooster H., Paepe D., Van der Meeren S., Dewulf J. (2018). Antimicrobial Prescribing Behaviour in Dogs and Cats by Belgian Veterinarians. Vet. Rec..

[45] Bäumer W., Merle R., Feuer L., Frenzer K., Lübke-Becker A. (2025). Resistance Situation of Selected Small Animal Pathogenic Bacteria and Prudent Use of Antibiotics—How Do I Integrate This?. Tierarztl. Prax. Ausg. K Kleintiere. Heimtiere..

[46] European Medicines Agency Committee for Medicinal Products for Veterinary Use (CVMP). https://www.ema.europa.eu/en/committees/committee-veterinary-medicinal-products-cvmp.

[47] Moja L., Abbas M., de Kraker M.E.A., Zanichelli V., Ombajo L.A., Sharland M., Huttner B. (2025). Reserve Antibiotics: Overcoming Limitations of Evidence Generated from Regulatory Approval Trials. Glob. Health.

[48] Wise M.G., Karlowsky J.A., Mohamed N., Hermsen E.D., Kamat S., Townsend A., Brink A., Soriano A., Paterson D.L., Moore L.S.P. (2024). Global Trends in Carbapenem- and Difficult-to-Treat-Resistance among World Health Organization Priority Bacterial Pathogens: ATLAS Surveillance Program 2018–2022. J. Glob. Antimicrob. Resist..

[49] Austin D.J., Kristinsson K.G., Anderson R.M. (1999). The Relationship between the Volume of Antimicrobial Consumption in Human Communities and the Frequency of Resistance. Proc. Natl. Acad. Sci. USA.

[50] Hao Y., Chen S., Chang H., Yan X., Zhou W., Cao X., Huang R., Zhang Z., Zhang H., Jia B. (2020). Temporal Association between Carbapenems Usage and Antimicrobial Resistance in Gram-Negative Bacteria at a Tertiary Hospital in Nanjing, China. Diagn. Microbiol. Infect. Dis..

[51] Pfeifer N.M., Weber M., Wiegand E., Barth S.A., Berens C., Menge C. (2025). Escherichia Coli Resistant to the Highest Priority Critically Important Fluoroquinolone or 3rd and 4th Generation Cephalosporin Antibiotics Persist in Pigsties. Appl. Environ. Microbiol..

[52] Sevilla-Navarro S., Catalá-Gregori P., Torres-Boncompte J., Orenga M.T., Garcia-Llorens J., Cortés V. (2022). Antimicrobial Resistance Trends of Escherichia Coli Isolates: A Three-Year Prospective Study of Poultry Production in Spain. Antibiotics.

[53] JIACRA III—Antimicrobial Consumption and Resistance in Bacteria from Humans and Animals. https://www.ecdc.europa.eu/en/publications-data/third-joint-interagency-antimicrobial-consumption-and-resistance-analysis-report.

